# An engineered CD81‐based combinatorial library for selecting recombinant binders to cell surface proteins: Laminin binding CD81 enhances cellular uptake of extracellular vesicles

**DOI:** 10.1002/jev2.12139

**Published:** 2021-09-12

**Authors:** Stefan Vogt, Madhusudhan Reddy Bobbili, Gerhard Stadlmayr, Katharina Stadlbauer, Jørgen Kjems, Florian Rüker, Johannes Grillari, Gordana Wozniak‐Knopp

**Affiliations:** ^1^ acib GmbH (Austrian Centre of Industrial Biotechnology) Graz Austria; ^2^ Department of Biotechnology Institute of Molecular Biotechnology University of Natural Resources and Life Sciences (BOKU) Vienna Austria; ^3^ Ludwig Boltzmann Institute for Experimental and Clinical Traumatology in the AUVA Research Center Vienna Austria; ^4^ Department of Biotechnology Christian Doppler Laboratory for Innovative Immunotherapeutics University of Natural Resources and Life Sciences (BOKU) Vienna Austria; ^5^ Department of Molecular Biology and Genetics Centre for Cellular Signal Patterns (CellPat) Interdisciplinary Nanoscience Centre (iNANO) Aarhus University Aarhus C Denmark

**Keywords:** antigen‐binding CD81, directed evolution, extracellular vesicle uptake, targeted extracellular vesicles, yeast display

## Abstract

The research of extracellular vesicles (EVs) has boomed in the last decade, with the promise of them functioning as target‐directed drug delivery vehicles, able to modulate proliferation, migration, differentiation, and other properties of the recipient cell that are vital for health of the host organism. To enhance the ability of their targeted delivery, we employed an intrinsically overrepresented protein, CD81, to serve for recognition of the desired target antigen. Yeast libraries displaying mutant variants of the large extracellular loop of CD81 have been selected for binders to human placental laminin as an example target. Their specific interaction with laminin was confirmed in a mammalian display system. Derived sequences were reformatted to full‐length CD81 and expressed in EVs produced by HeLa cells. These EVs were examined for the presence of the recombinant protein and were shown to exhibit an enhanced uptake into laminin‐secreting mammalian cell lines. For the best candidate, the specificity of antigen interaction was demonstrated with a competition experiment. To our knowledge, this is the first example of harnessing an EV membrane protein as mediator of de novo target antigen recognition via in vitro molecular evolution, opening horizons to a broad range of applications in various therapeutic settings.

**Abbreviations**
BACE1: beta‐secretase 1
BCA: bicinchoninic acid
BSA: bovine serum albumin
cel‐miR‐39: *Caenorhabditis elegans micro*‐RNA‐39Ct cycle threshold
DMEM: Dulbecco's modified Eagle medium
eGFP: enhanced green fluorescent protein
EGFR: epithelial growth factor receptor
EV: extracellular vesicle
Fab: fragment antigen binding
FACS: fluorescence‐activated cell sorting
FBS: fetal bovine serum
FITC: fluorescein isothiocyanate
FSC: forward scatter
HA: hemagglutinin
LEL: large extracellular loop
MACS: magnetic‐activated cell sorting
MES: 2‐(N‐morpholino) ethanesulfonic acid
MFI: mean fluorescence intensity
miRNA: microRNA
MWCO: molecular weight cut‐off
NTA: nanoparticle tracking analysis
PBS: phosphate buffered saline
PBS‐T: PBS with 0.1% Tween‐20
PCR: polymerase chain reaction
PE: phycoerythrin
PEG: polyethyleneglycol
PEI: polyethyleneimine
PVDF: polyvinylidene difluoride
qPCR: Quantitative reverse transcription‐PCR in real time
RIPA: radioimmunoprecipitation assay
RT: room temperature
SDS: sodium dodecylsulphate
SEL: small extracellular loop
siRNA: small interfering RNA
SSC: side scatter
TEM: transmission electron microscopy
T_M_
: midpoint of thermal denaturation

## 1 INTRODUCTION

In recent years, extracellular vesicles (EVs) have gained recognition as elaborate drug delivery vehicles (Yamamoto et al., 2019). These 50–150 nm large membrane enveloped structures, originating from the lamellae of the multivesicular body, have been discovered as cell‐to‐cell messengers, as their contents, composed of a cocktail of proteins, miRNAs and other non‐coding RNAs, originating from the producing cell, can efficiently be transferred to a recipient cell upon fusion with its cellular membrane (Yáñez‐Mó et al., 2015), by endocytosis and phagocytosis. EVs have been shown to play a vital role in intercellular communication in several biological situations crucial for sustaining homeostasis in tissues and organs (H. Rashed et al., 2017), aging and senescence (Terlecki‐Zaniewicz et al., 2019; Weilner et al., 2016), and further, to support processes such as regeneration after injury (Taverna et al., 2017) and limitation of tumorigenesis and malignant transformation (Wolfers et al., 2001). These properties have rendered them interesting for biotechnological applications, which has resulted in accelerated development of methods for their production, including optimization of source cells and respective culture media (Patel et al., 2018; Roura & Bayes‐Genis, 2019), improved purification processes (Zhang et al., 2019), standardization of protocols for characterization (Coumans et al., 2017) and stable storage of EV preparations (Jeyaram & Jay, 2018). It was soon accepted that exogenous loading of EVs with biologically diverse compounds, such as siRNAs, miRNAs, genes and toxins, can be applied for enhanced delivery of the desired substance into target cells (Batrakova & Kim, 2015). Sophisticated protocols have been devised to enable optimal enclosure of the drug of interest into the vesicles (Sutaria et al., 2017), employing a spectrum of strategies, including simple diffusion, lipofection, electroporation and sonication and endogenous loading methods related mostly to RNA‐cargo (overexpression in host cells (Hung & Leonard, 2015), extrusion of host cells (Lunavat et al., 2016), vexosome hybrid vehicles (Maguire et al., 2012) and introduction of EXOmotif to bias the miRNA loading into the EVs (Villarroya‐Beltri et al., 2013)).

On the other hand, targeted delivery of EVs has long been recognized as a feature that could crucially improve their biological effect. The membrane composition of the native EVs is unique as it exhibits substantial variation depending strongly on the cell of origin (Yoshioka et al., 2013) and assigning them a level of tropism for target cells (Hoshino et al., 2015). Nevertheless, the first experiments with EVs in vivo, especially those including intravenous applications, have shown that such agents could profit from improved specific delivery when their surface has been modified to enable the specific recognition of the target cells (Walker et al., 2019). Prominent examples featuring surface modification of EVs as beneficial for delivery of therapeutic compounds include overexpression of a surface‐bound peptide recognizing specifically the tumour marker EGFR, which led to a higher level of accumulation of modified vesicles in target cells, and in addition such particles loaded with let‐7a miRNA were able to inhibit tumour growth in mice (Ohno et al., 2013). Derivatization of an endogenously overrepresented EV surface protein to achieve enhanced targeted uptake was validated as a strategy with experiments where such particles were able to efficiently deliver anti‐BACE1 siRNA to the brain in mice (Alvarez‐Erviti et al., 2011). Targeted delivery could also be achieved via fusion with immunoglobulin‐based antigen‐binding molecules, such as single chain antibodies (Wang et al., 2018) and nanobodies (Kooijmans et al., 2016). Chemical strategies on the other hand involve ex vivo membrane modification, as presented for example for the ‘cloaking’ platform where streptavidin‐decorated EV surfaces can be endowed with a biotinylated targeting agent of choice (Antes et al., 2018). To sum up, derivatization of EV surface structures appears to be a viable strategy to enhance their targeted uptake, and the diverse spectrum of methods to achieve this goal is yet to be explored.

For this purpose, we have opted to leverage the innately overrepresented EV surface protein CD81, which is at the same time regarded as their hallmark surface marker, into an antigen‐recognition scaffold. This protein is a member of the tetraspanin family, which features four transmembrane segments (TM1–4) linked by a small intracellular loop, a small extracellular loop (SEL) and a large extracellular loop (LEL) (Levy et al., 1991). The crystallographic structure of a soluble form of the tetraspanin CD81 LEL domain is a five‐helix bundle stabilized by two disulfide bridges (Kitadokoro, 2001; Kitadokoro et al., 2001). The protein fold as well as its key structural features are conserved among tetraspanins, while a high degree of variability in its length and composition can be found in its central part that in CD81 comprises three helices (Seigneuret, 2006; Seigneuret et al., 2001) Previously, we have designed highly stabilized mutants of CD81 LEL via introduction of de novo disulfide bonds (Vogt et al., 2018). Two such CD81 LEL variants, the most stable one and the variant that exhibited reversible refolding after denaturation, were chosen to serve as scaffolds for libraries of antigen‐binding units, obtained by randomization of selected solvent‐exposed residues. The selection platforms were constructed using yeast display as a validated method of molecular evolution in vitro and were envisioned as a source of specific binders to an antigen of choice.

One of the most quickly expanding therapeutic areas that could profit from use of targeted EVs is regeneration of a variety of tissues, including bone (Inder et al., 2014; Qin et al., 2016), tendons (Wang et al., 2019), cartilage (Tan et al., 2020), skin (Terlecki‐Zaniewicz et al., 2019; Wu et al., 2018) and importantly neural tissue (Ching & Kingham, 2015; Dong et al., 2019), whose repair is complex depending on processes involving changes in Schwann cell phenotypes, the reprogramming of macrophages, and the reconstruction of the vascular network critically dependent on paracrine secretion (Cattin et al., 2015). Laminin can efficiently support the process of muscular regeneration (Riederer et al., 2015) and neural regeneration as it induces rapid and efficient adhesion of primary Schwann cells and vascular tube formation by endothelial cells (Hackethal et al., 2018). Therefore, we selected this protein as a target antigen for selection of specifically binding CD81 LEL mutants from a yeast library. To improve the discovery of hits that could function as recognition agents on the surface of EVs, we tested the antigen‐binding CD81 LEL variants in a mammalian display system and putative candidates were then expressed as full‐length CD81 in HeLa cells. To assess their novel functionality, internalization of such EVs into laminin‐secreting cell lines Huh‐7 and NCI‐N87 was compared with EVs derived from wild‐type CD81‐transfected production cell line, and further, their reactivity with several recombinant laminin isoforms was examined. The specificity of binding of laminin targeting EVs to their cognate antigen was tested under competitive conditions. Finally, the ability of laminin‐targeting EVs to transfer cel‐miR‐39 to Huh‐7 cell line was examined.

## 2 MATERIALS AND METHODS

### 2.1 Molecular design

The CD81 crystal structure PDB: 1G8Q was used as the basis of selection of solvent‐exposed residues that could upon randomization form a novel antigen binding site of about 600 Å^2^ in size. The first scaffold molecule was a CD81 LEL mutant that harbours two novel disulfide bonds, Ala134Cys/Lys144Cys and Val135Cys/Ser168Cys, and exhibits the midpoint of thermal transition (T_M_) of 109.4°C, 43°C above that of the wild‐type CD81 LEL. The library design named CD81LEL_2 involved randomization of 13 amino acid residues: 132–133, 136–139, 162–165, 167 and 171–172 (numbering as in PDB: 1G8Q). CD81LEL_L3 library design was based on the reversibly refolding CD81 LEL mutant, where the stabilizing mutations Ala130Cys/Ala146Cys and Val135Cys/Ser168Cys work additively to increase T_M_ of CD81 LEL to 93.4°C, and render the mutated CD81 LEL the ability to reversibly refold when heated up to 110°C, unlike the wild‐type CD81 LEL. In this library, the amino acid residues at the positions 132–133, 136–141, 162–165, 167 and 171–172 were randomized to form a library of composite surfaces available for antigen binding.

### 2.2 Yeast display

#### 2.2.1 Preparation of yeast display libraries

For validation of feasibility of yeast display of CD81 scaffold, CD81 LEL domain sequence (synthetized by Geneart, Thermo Fisher Scientific) was cloned into the pYD1 vector (Thermo Fisher Scientific) and transformed into *S. cerevisiae* EBY100 (Thermo Fisher Scientific) using the PEG3350/Li‐acetate/salmon sperm DNA protocol (Gietz & Schiestl, 2007). Selection, cultivation and induction of transformed colonies proceeded in S‐CAA media containing either 2% glucose (D) or 2% galactose/1% raffinose (G/R) as carbon source, according to published protocols (Chao et al., 2006). The recipient vector for the yeast display library was modified by deletions and insertions of restriction enzyme recognition sites using Quikchange Lightning Mutagenesis Kit (Agilent) to simplify linearization for use in gap‐repair driven homologous recombination. Oligonucleotides for yeast library construction were synthetized by ELLA Biotech. PCR recombination products encoding the randomized inserts were produced using in 100 μl aliquots using Q5 HiFi Polymerase MasterMix (New England Biolabs), 10 ng/μl template DNA encoding CD81 LEL, and 50 pmol of each of oligonucleotides L2for and EFrev for the library CD81LEL_L2, or 50 pmol of each of oligonucleotides L3for and EFrev (sequences of all oligos in the Table S1). The size of the yeast display libraries, which were produced each in two batches labelled A and B, was determined using dilution plating. To investigate the level of correctness, plasmid DNA was isolated from 10 μl of pelleted yeast cells using Zymoprep II kit (Zymo Research) and transformed to *E. coli* TOP10 using electroporation. The expression cassettes of CD81 LEL mutants were amplified using primers pyd forward and pyd reverse (Thermo Fisher Scientific) and sequenced using one of these primers.

#### 2.2.2 Staining of yeast‐display libraries for FACS‐based phenotype control

For quality control, the yeast cells were induced in SG/RCAA medium with penicillin and streptomycin either for 48 h at 20°C or 24 h at 37°C. For staining, induced yeast cells were blocked in 2% BSA‐PBS solution for 30 min at RT at an OD_600_ of 1. Then they were resuspended into 100 μl‐aliquots and stained with anti‐Xpress antibody (R910‐25, Thermo Fisher Scientific) (1:1000) reactive with the N‐terminally positioned Xpress tag, and M38 antibody (10630D, Thermo Fisher Scientific) (1 μg/ml), which detects the properly folded CD81 LEL (Imai & Yoshie, 1993), in 2% BSA‐PBS for 1 h at RT. Cells were pelleted at 1000 × *g*, for 5 min at 4°C and resuspended in 2% BSA‐PBS with goat anti‐mouse (Fab’)_2_‐FITC conjugate (F‐2653, Sigma‐Aldrich), diluted 1:200. Samples stained with anti‐his‐tag‐Alexa Fluor 488 antibody (35351, QIAgen), diluted 1:200 in 2% BSA‐PBS and anti‐V5‐tag‐FITC antibody (MA1‐80281, Thermo Fisher Scientific), diluted 1:100 in 2% BSA‐PBS, were used to detect C‐terminally positioned tags to determine the percentage of read‐through sequences of the clones. The incubation time with fluorescent antibodies was 30 min on ice. The fluorescence of stained samples and unstained controls was determined using Guava easyCyte flow cytometer (Merck Millipore).

#### 2.2.3 Selection of yeast display libraries with human placental laminin

Human placental laminin was purchased from Sigma‐Aldrich. For biotinylation, EZ‐Link Sulfo‐NHS‐LC‐LC‐biotin reagent (Thermo Fisher Scientific) was used in a 3:1 molar ratio, after dialysis of the antigen against 100‐fold volume of PBS overnight at 4°C. Incubation with biotinylation reagent proceeded for 1 h at RT with shaking. Unbound biotin was removed by dialysis against 100‐fold volume of PBS, using Snakeskin dialysis tubing with 10,000 Da MWCO (Thermo Fisher Scientific), at 4°C overnight with stirring.

Yeast display libraries L2A, L2B, L3A and L3B were cultured in SD‐CAA medium and the display of recombinant protein was induced by incubating them in SG/R‐CAA medium with shaking overnight at 37°C. For MACS, 10^9^ induced yeast cells of each library were selected for binding to 1 μM biotinylated antigen and captured onto streptavidin microbeads (μMACS streptavidin kit, MACS Miltenyi), exactly according to manufacturer's instructions. In FACS selection rounds, the induced cell suspensions were diluted to 10^8^ cells per ml 10% BSA‐PBS and labelled with 1 μM biotinylated laminin and streptavidin‐Alexa Fluor 647 (1:800) (S21374, Thermo Fisher Scientific) and anti‐V5‐FITC antibody (1:100) (MA1‐80281, Thermo Fisher Scientific) as secondary reagents. In all FACS‐assisted selection rounds, at least 20 times output of the previous sorting round was processed and 0.1% top anti‐V5‐antibody positive yeast cells were collected with a Sony SH8000 cell sorter. After visible enrichment, collected cells were plated out to characterize single yeast display clones. Yeast pools enriched for antigen binders entered an additional selection round using 200 nM antigen.

#### 2.2.4 Analysis of antigen binding of CD81 LEL clones in mammalian cell display

The sequences of laminin binding clones were cloned between the *Sfi*I and *Sal*I restriction sites of the pDisplay vector (Thermo Fisher Scientific) that allows the expression of C‐terminally anchored protein of interest between N‐terminal HA‐tag and C‐terminal c‐myc‐tag. Plasmids encoding CD81 LEL mutants were introduced into 2‐ml‐cultures of HEK293‐6E cells (Canadian National Research Council) using PEI‐mediated transfection exactly according to manufacturer's instructions and the expression proceeded for 48 h on an orbital shaker at 180 rpm in humidified atmosphere with 5% CO_2_ at 37°C. After expression, the transfected cells were harvested at 300 × *g*, 10 min at 4°C, blocked for 30 min in 2% BSA‐PBS on ice. For the wild‐type CD81 LEL, the number of copies on the mammalian cell surface was determined using QIFIKIT (Agilent) and an anti‐c‐myc antibody 9E10 (Calbiochem) at 20 μg/ml, and the proper folding of the displayed CD81 LEL was confirmed with staining with M38 antibody (10630D, Thermo Fisher Scientific) at 10 μg/ml, followed by staining with anti‐mouse (Fab’)_2_‐FITC conjugate (F‐2653, Sigma‐Aldrich), diluted 1:200 in 2% BSA‐PBS. HEK cells expressing CD81 variants were stained with an anti‐c‐myc antibody (A‐14, sc789, Santa Cruz) at 10 μg/ml in 2% BSA‐PBS, for 30 min on ice. Binding was detected after incubation with anti‐rabbit IgG (H+L) ‐ Alexa Fluor 488 (A‐11034, Thermo Fisher Scientific), diluted 1:1000 in 2% BSA‐PBS, for 30 min on ice. Antigen reactivity was determined after incubation with 500 nM biotinylated human laminin and detection with streptavidin‐Alexa Fluor 647 (S21374, Thermo Fisher Scientific) at 1:1000 in 2% BSA‐PBS with Guava easyCyte flow cytometer (Merck Millipore).

### 2.3 EV preparation and characterization

#### 2.3.1 Cell culture

HeLa cells (ATCC CCL‐2) were cultured at 37°C in 5% CO_2_ in RPMI 1640 medium (Sigma‐Aldrich), supplemented with 10% foetal bovine serum (FBS) and 4 mM L‐glutamine (Thermo Fisher Scientific) and used at passage 2–30. These cells were transduced with pBMN‐I‐GFP (Addgene)‐based constructs encoding full‐length CD81 variants with C‐terminal eGFP fusion (cloning details in File S1) using the retroviral Phoenix‐AMPHO transduction system. Phoenix‐AMPHO cells (ATCC CRL‐3213) were cultured in DMEM with 10% FBS and 4 mM L‐glutamine. Virus particle generation was performed by transfecting Phoenix‐AMPHO cells at 80 % confluency using jetPRIME (Polyplus Transfection) according to the manufacturer's brochure. Supernatants containing virus particles were filtered through a 0.45 μm PVDF filter and added to the HeLa cells. Transduced cells were sorted for strongly eGFP‐expressing variants with a Sony‐SH8000 sorter. To compare the expression of recombinant CD81‐eGFP variants, cells were harvested with trypsinization and the level of green fluorescence in a flow cytometry‐based assay was examined. Additionally, localization of the constructs was examined at cellular level using live‐cell microscopy (experimental details in File S1).

#### 2.3.2 Isolation of extracellular vesicles

HeLa cells stably expressing CD81‐eGFP variants were cultured for 24–48 h. When cells reached a confluency of 75–85%, growth medium was discarded and replaced by OptiMEM reduced serum medium (Thermo Fisher Scientific), which was collected after 48 h when the cells were 95%–98% confluent with a viability over 95% as determined with Trypan blue exclusion method using the Vi‐CELL XR Counter (Beckman Coulter). The conditioned medium was centrifuged at 700 g (5804R; Eppendorf, Hamburg, Germany) for 5 min at 4°C to exclude cells and cellular debris. The supernatant was further centrifuged at 2000 × *g* (5804R; Eppendorf, Hamburg, Germany) for 10 min at 4°C to exclude larger agglomerated particles. After filtration through a 0.2‐μm PVDF‐filter, the supernatant was filled into polycarbonate 70‐ml 38 × 102 mm tubes (Beckman Coulter) to enrich EVs at 125,000 × *g* for 90 min at 4°C using a 45 Ti Rotor (Beckmann Coulter). The pellets were then re‐suspended in 1.5 ml of Live Cell Imaging Solution (Thermo Fisher Scientific), pH 7.4, per tube, and the ultracentrifugation step was repeated to obtain an EV pellet that was washed once, re‐suspended in 200–1000 μl of the same buffer and stored at ‐80°C until further analysis.

Where indicated, EV preparations were additionally treated: after ultracentrifugation, they were resuspended in 50 μl PBS and incubated for 45 min at 37°C with 550 μl of TrypLE‐Select agent (Thermo Fisher Scientific), to digest residual matrix that could contain laminin fragments and to reduce the EVs’ corona. The effect of TrypLE‐Select enzyme on the integrity of cell surface‐expressed CD81 was examined in comparison with 0.05% trypsin solution that is traditionally used (experimental details in File S1).

#### 2.3.3 Nanoparticle tracking analysis (NTA)

The median, mean and mode sizes of extracellular vesicles as well as their concentration and size distribution were determined by nanoparticle tracking analysis on a ZetaView BASIC PMX‐120 (Particle Metrix GmbH). ZetaView software (version 8.05.11 SP4) was used to determine particle count, size parameters and distribution. The ZetaView device was calibrated with supplied standard beads. EV samples were diluted in PBS for further analysis. Particle measurements were captured and evaluated in three replicates.

#### 2.3.4 Western blot

EV pellets isolated by ultracentrifugation were directly resuspended in RIPA buffer (50 mM Tris‐HCl, pH 8.0, with 150 mM sodium chloride, 1.0% Igepal CA‐630 (NP‐40), 0.5% sodium deoxycholate, and 0.1% sodium dodecyl sulfate), kept on ice, and vortexed five times every 5 min. HeLa cells were collected and the cell pellet was lysed with 100 μl of RIPA buffer, kept on ice, and vortexed five times every 5 min. The cell lysate was then spun at 12,000 × *g* for 10 min at 4°C and the supernatant was transferred to a new tube and kept on ice. Protein concentrations for the cell lysate supernatants and EV lysates were quantified using BCA assay (Thermo Fisher Scientific) according to manufacturer's instructions.

Thirty micrograms of cell lysate and 15 micrograms of EVs were mixed with buffer containing 0.5 M dithiothreitol, 0.4 M sodium carbonate (Na_2_CO_3_), 8% SDS, and 10% glycerol, and heated at 95°C for 10 min. The samples were loaded onto a NuPAGE Novex 4–12% Bis‐Tris Protein Gel (Invitrogen, Thermo Fisher Scientific) and run at 120 V in NuPAGE MES SDS running buffer (Invitrogen, Thermo Fisher Scientific) for 2 h. The proteins on the gel were transferred to a Trans‐Blot Turbo Mini PVDF Transfer for 7 min using the Trans Blot Turbo Blotting System (Bio‐RAD) apparatus. The membrane was blocked with 3% BSA in PBS with 0.1% Tween‐20 (PBS‐T, Sigma‐Aldrich), for 1 h with gentle shaking. After blocking, the membrane was incubated overnight at 4°C with primary antibody solution: anti‐syntenin at 1:1000 (TA504796, Origene, mouse monoclonal), anti‐TSG101 at 1:1000 (ab125011, Abcam, rabbit monoclonal), anti‐Alix at 1:2000 (ab117600, Abcam, mouse monoclonal), or anti‐calnexin at 1:1000 (ab22595, Abcam, rabbit polyclonal). The membrane was washed with PBS‐T three times for 5 min and incubated with the corresponding secondary antibody: donkey anti‐mouse‐IRDye 680RD at 1:10,000 (926‐68072, LI‐COR) or donkey anti‐rabbit‐ IRDye 800CW at 1:10,000 (925‐32213, LI‐COR), for 1 h at RT. Finally, the membrane was washed three times for 5 min with PBS‐T, twice for 5 min with PBS and visualized on the Odyssey infrared imaging system (LI‐COR) at 700 and 800 nm.

#### 2.3.5 Transmission electron microscopy (TEM)

Samples of isolated EVs were pre‐diluted in Live Cell Imaging Solution (Thermo Fisher Scientific) and transferred to copper grids which were previously glow, discharged for 45 s and incubated for 1 min, followed by careful blotting. Uranyl formate solution (2% in 20 mM KOH) was delivered initially for 5 s, then discarded and used again for a 30‐s‐incubation. The staining solution was blotted and fully dried on air. Imaging was performed by using a Tecnai G2 Spirit electron microscope (FEI Company) operated at 120 kV and images were acquired by a CMOS 4k camera (TVIPS GmbH).

#### 2.3.6 Uptake of targeting EVs

Hepatocarcinoma cell line Huh‐7 (JCRB0403, NIBIOHN) and gastric adenocarcinoma cell line NCI‐N87 (ATCC CRL‐5822) were maintained at 37°C in 5% CO_2_ in high glucose DMEM (Thermo Fisher Scientific), supplemented with 10% FBS (Sigma‐Aldrich), and 4 mM L‐glutamine (Thermo Fisher Scientific). Their secretion of laminin was visualized as described in the File S1. Per well, 1.5 × 10^5^ Huh‐7 and 2 × 10^5^ NCI‐N87 cells were seeded into 24 well‐plates and allowed to attach overnight. Medium was then replaced with 300 μl of OptiMEM reduced serum medium containing graded concentrations of 2.5, 1.25 and 0.75 × 10^10^ recombinant EVs harbouring CD81‐eGFP and the antigen‐targeting variants, and incubated for 4 h at 37°C. Cells were then detached with 0.1% trypsin‐EDTA (Thermo Fisher Scientific) for 5 min at 37°C, neutralized using 1x Trypsin Inhibitor (Thermo Fisher Scientific) and resuspended in 200 μl PBS. Flow cytometry was used to measure the uptake levels of eGFP‐positive EVs in that the MFI values of the main cell population, gated out in the FSC/SSC plot, were determined using a CytoFLEX S instrument (Beckman Coulter). The fluorescence of the untreated cells was subtracted from all readings. Fluorescence relative to the cells treated with wild‐type CD81 expressing EVs was calculated.

### 2.4 Transfer of cel‐miR‐39 by targeting EVs

#### 2.4.1 Cel‐miR‐39 transfection

HeLa cells stably expressing CD81‐eGFP variants were reverse‐transfected with *C. elegans* specific cel‐miR‐39 precursor (AM17103, Thermo Fisher Scientific) using siPORT NeoFX transfection agent (AM4511, Thermo Fisher Scientific) according to the manufacturer's protocol. In brief, per well, 5 μl of siPORT NeoFX transfection agent and 7.5 μl of 10 μM pre‐cel‐miR‐39 were diluted in 100 μl of Opti‐MEM medium separately, then mixed and incubated for 10 min at room temperature. RNA/siPORT NeoFX Transfection Agent transfection complexes were dispensed into wells of a 6‐well‐culture plate containing 3 × 10^5^ HeLa cells stably expressing CD81‐eGFP variants. These were incubated for 24 h at 37°C in 5% CO_2_, and then the medium was replaced with Opti‐MEM reduced serum medium and the incubation continued for 48 h. All the cel‐miR‐39 transfer studies were performed as before (Hill et al., 2020; Terlecki‐Zaniewicz et al., 2019).

#### 2.4.2 EV isolation by ultrafiltration

The conditioned medium after 48‐h‐incubation was centrifuged at 700 × *g* for 5 min at 4°C to exclude cells and cellular debris. The supernatant was further centrifuged at 14,000 × *g* for 15 min at 4°C to exclude larger agglomerated particles. EVs in the conditioned medium were concentrated to 100 μl using Amicon Ultra‐0.5 centrifugal filter unit (UFC501096) at 14,000 × *g* at 4°C. EVs, secreted by 1 × 10^6^ cells, were used for functional uptake studies in Huh‐7 cells. In brief, 3 × 10^5^ Huh‐7 cells were seeded into 6‐well‐plates and allowed to attach overnight. Medium was then replaced with 1 ml of Opti‐MEM reduced serum medium containing recombinant EVs harbouring CD81‐eGFP or the antigen‐targeting variants produced by cel‐miR‐39‐transfected cells, and incubated for 24 h at 37°C. Where indicated, EV preparations were additionally treated with 1% Triton X‐100 and 38 μg/ml RNase A (Thermo Fisher Scientific) in PBS for 30 min at 37°C and afterwards washed using Amicon Ultra‐0.5 centrifugal filter unit (UFC501096) at 14,000 × *g* at 4°C, with the purpose to demonstrate that the cel‐miR‐39 is encapsulated in EVs.

#### 2.4.3 RNA isolation

Transfected HeLa cells after EV harvest, and Huh‐7 cells after incubation with EVs carrying cel‐miR‐39, were lysed in TRIreagent (Sigma‐Aldrich) and small RNA isolation was performed using miRNeasy Mini kit (QIAgen) according to manufacturer's instructions. RNA concentration and quality were assessed using Nanodrop spectrometer (ND‐ONE‐W; Thermo Fisher Scientific).

#### 2.4.4 cDNA synthesis

Ten ng of small RNA was used for cDNA synthesis using miRCURY LNA RT kit (QIAgen). cDNA was synthetized at 42°C for 60 min, followed by enzyme inactivation step for 5 min at 95°C. Specific synthesis reaction for UniSp6 used as a spike‐in was included in all samples as a control for enzyme activity.

#### 2.4.5 Quantitative reverse transcription‐PCR in real time (qPCR)

miRNA qPCR analysis was performed using miRCURY LNA SYBR Green PCR kit (QIAgen) and LNA‐optimized primers. Primers used for qPCR are as follows: cel‐miR‐39, cel‐miR‐39‐3p miRCURY LNA miRNA PCR Assay (GeneGlobe ID: YP00203952; 339306; QIAgen); UniSp6, UniSp6 miRCURY LNA miRNA PCR Assay (GeneGlobe ID: YP00203954; 339306; QIAgen). All experiments were performed with a Rotor‐Gene Q cycler.

For all subsequent steps, the raw Ct values were normalized to total viable cell number for each sample. For better visualization, Ct values were further transformed to arbitrary units by assuming a Ct value of 45 to be 1 arbitrary unit (arbitrary units are presented as absolute values).

Statistical analysis was performed using one‐way ANOVA with Graph Pad Prism program, version 5.03.

#### 2.4.6 Binding of recombinant EVs to laminin variants

Biotinylated recombinant laminin variants Lam521, 511, 421 and 332 and EHS‐laminin111 (Biolamina, Sundbyberg, Sweden) were used in bead‐based laminin‐binding assay. 2 × 10^10^ EVs were added to 4 × 10^3^ CD9 Exosome Capture Beads (Immunostep) in a total volume of 80 μl 3% BSA‐PBS for 3 h at RT. Beads were collected with centrifugation at 3000 × *g* for 5 min at RT and washed once with 100 μl PBS. Five μg/ml of biotinylated laminin variants in 3% BSA‐PBS were added to the EV‐bead mixture and incubated for 30 min at RT. After pelleting and a washing step with 3% BSA‐PBS at 4°C, neutravidin‐PE (Thermo Fisher Scientific) (1:800) in 3% BSA‐PBS was added for 30 min on ice to detect EV‐bound laminin in a flow cytometry measurement performed on CytoFLEX S instrument (Beckman Coulter). Median fluorescence values of FSC/SSC gated population were expressed as multiple of readings obtained with wild‐type CD81 EVs after values obtained with the secondary reagent alone were subtracted.

#### 2.4.7 Specificity of laminin binding of recombinant EVs

Five times 10^9^ recombinant EVs harbouring wild‐type CD81‐eGFP or its laminin‐targeting variant L2ALU_1 were incubated with 6 × 10^3^ CD9 Exosome Capture Beads (Immunostep) in 120 μl 3% BSA‐PBS for 3 h at RT. Beads were collected with centrifugation at 3000 × *g* for 5 min at RT and washed once with 100 μl PBS. Five μg/ml of biotinylated human placental laminin in 3% BSA‐PBS, and additionally 15 μg/ml of unlabelled laminin for competition in parallel samples were added to the EV‐bead mixture and incubated for 30 min at RT. After pelleting and a washing step with 3% BSA‐PBS at 4°C, neutravidin‐PE (Thermo Fisher Scientific) (1:800) in 3% BSA‐PBS was added for 30 min on ice to detect EV‐bound laminin in a flow cytometry measurement performed on CytoFLEX S instrument (Beckman Coulter).

## 3 RESULTS

### 3.1 Selection of CD81 LEL variants with antigen‐binding activity

**FIGURE 1:**
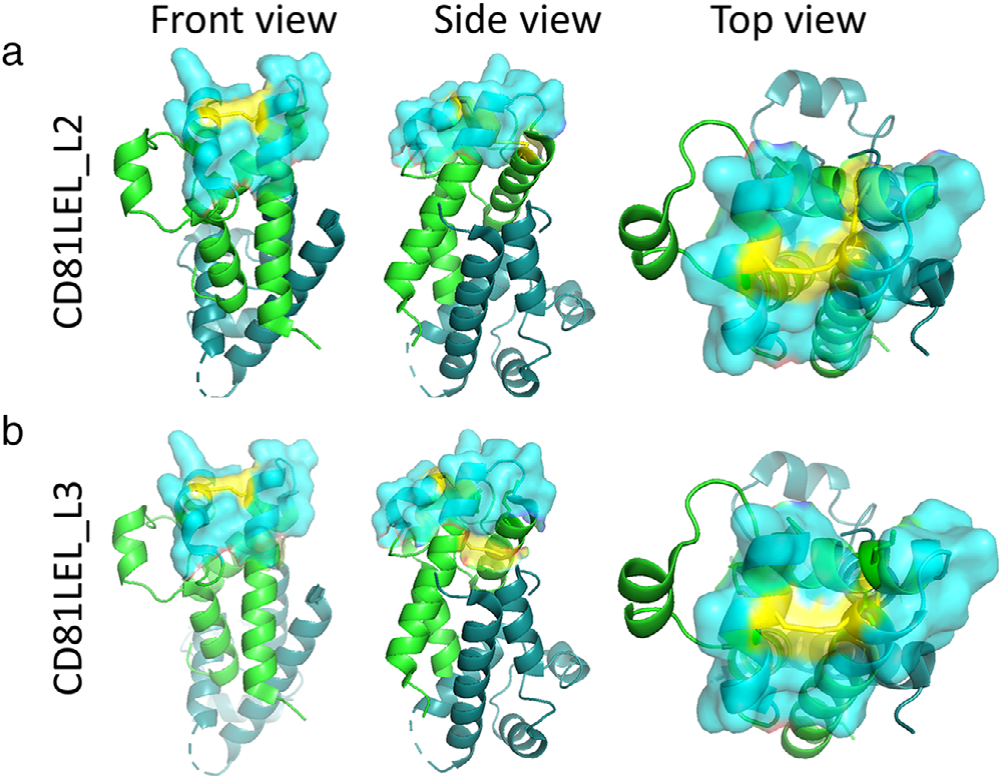
CD81 LEL library designs with de novo disulfide bridges (yellow) and mutated amino acid residues (cyan, surfaced) on protomer a (green), protomer b is in teal: (a) CD81LEL_L2; (b) CD81LEL_L3, front view (left panel), side view (centre panel) and side view (right panel). The figure is based on PDB: 1G8Q using The PyMOL Molecular Graphics System, Version 1.3, Schrödinger, LLC

**FIGURE 2:**
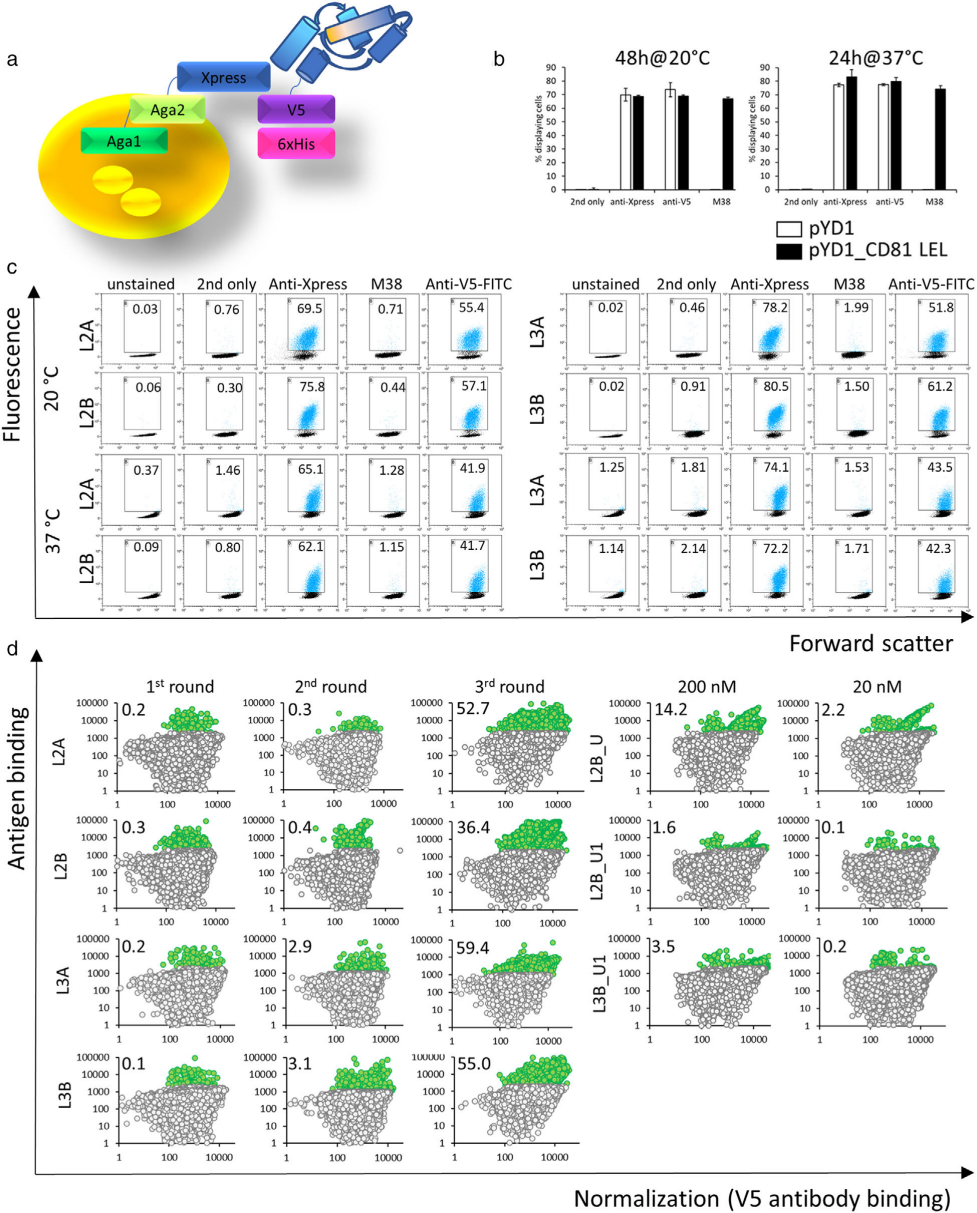
Yeast display of mutant CD81 LEL: (a) Schematic of yeast display; (b) Display of wild‐type CD81 LEL (black bars) in comparison with empty vector pYD1 (white bars), percentage of cells displaying N‐terminal Xpress tag, C‐terminal V‐tag and correctly folded CD81 LEL (M38‐antibody positive) are shown; (c) Quality control of libraries CD81LEL_L2 and CD81LEL_L3. Figures indicate the percentage of gated cells; (d) Results of sorting of CD81 LEL libraries for laminin binders. Figures indicate the percentage of antigen‐positive cells

Among the membrane‐overrepresented proteins considered markers for the EVs, CD81 has been prominent for a long time (Escola et al., 1998). Availability of several high‐resolution crystal structures has corroborated CD81 LEL as an independent folding unit (Cunha et al., 2018; Kitadokoro, 2001; Kitadokoro et al., 2001; Nelson et al., 2018). Previously, we have attempted to improve the biophysical properties of CD81 LEL and constructed mutants that exhibit in one case an extremely high level of thermostability and in one case reversible thermal denaturation (Vogt et al., 2018). As these should be more permissive for mutagenesis required to introduce a novel antigen binding site, we have identified amino acid residues that could form a novel antigen‐binding surface based on the above mutants. Addressed were the groups of residues that are distal from the cell membrane, to diminish the probability of steric occlusion upon the expression in the context of the full‐length CD81 on the surface of cells of EVs. Randomization of 13 amino acid residues in the library CD81LEL_L2 and 15 amino acid residues in CD81LEL_L3, which corresponds to 14.6% and 16.8% of the original CD81 LEL sequence, was hypothesized to lead to a postulated available antigen binding surface of about 600 Å^2^ (Figure 1). The decision to use yeast display for binder selection was based on efficient display of wild‐type CD81 LEL on the yeast surface, observed even after stress induction conditions at 37°C, where only properly folded proteins express well due to the stringent quality control system of yeast (Shusta et al., 2000). Over 70% of the cells were positive for binding of antibodies reactive with N‐terminal Xpress tag and C‐terminal c‐myc and his‐tags (Schematic in Figure 2a), and encouragingly a similar level of reactivity was observed with a structure‐dependent binding antibody M38 (Imai & Yoshie, 1993) (Figure 2b). The library sizes were determined to be 1.1 × 10^8^ independent members for CD81LEL_L2 and 1.2 × 10^8^ independent members for CD81LEL_L3, and the correctness of the library L2A was found to be 62.5%, L2B 87.5%, L3A 62.5% and L3B 100% as determined by single clone sequencing. The staining with C‐terminal tag‐reactive antibodies revealed at least 40% positive cells at stress induction conditions at 37°C, indicating that the libraries were suitable for antigen selection (Figure 2c).

Enrichment of antigen‐binding clones from all libraries was observed as a double‐positive population after MACS and two following FACS sorts and proceeded to a similar level for both library designs (Figure 2d). After the 3r sorting round different gates, U and U1, were set to improve the chance of discovery of unique clones: four such were identified from library 2 and four from library 3 (sequences in Table 1).

**TABLE 1:** Amino acid sequences of laminin‐binding yeast clones

LibraryAntigen concentration	AA residues	132‐133	136‐139	162‐165	167	171‐172
CD81LEL_L2	Wild type	QQ	VDDD	LTAL	T	KN
	Clone					
1000 nM	L2ALU_1	RE	NTFD	THSS	G	FF
	L2ALU1_1	MI	HRHY	PSST	P	QN
	L2BLU_1	VF	FGGH	HPYK	K	QR
	L2BLU1_1	WK	NSLQ	YLCA	Y	WP
200 nM	L1_1	VF	FGGH	HPYK	K	QR
	L1_13	FI	FRRY	RNNE	A	AK

	AA residues	132‐133	136‐141	162‐165	167	171‐172
CD81LEL_L3	Wild type	QQ	VDDDAN	LTAL	T	KN
	Clone					
1000 nM	L3ALU_1	LW	SKRYKY	MGNL	E	IE
	L3BLU_1	RG	RDFTGS	KLRA	L	IK
	L3BLU_2	KY	KQGDVE	VANM	R	PA
	L3BLU_4	SW	NRKYEP	KANY	H	EM
200 nM	L1_17	SW	NRKYEP	KANY	H	EM
	L1_21	RH	SGLSGR	IKHK	L	IK

Enriched pools were then stained with decreasing concentrations of antigen and a double positive population was still discernible with 200 nM biotinylated laminin for sorts L2B_U, L2B_U1, and L3B_U1 (Figure 2d). Single colonies were sequenced and two new binder sequences were discovered (Table 1).

### 3.2 Laminin‐binding CD81 LEL clones in mammalian yeast display

**FIGURE 3:**
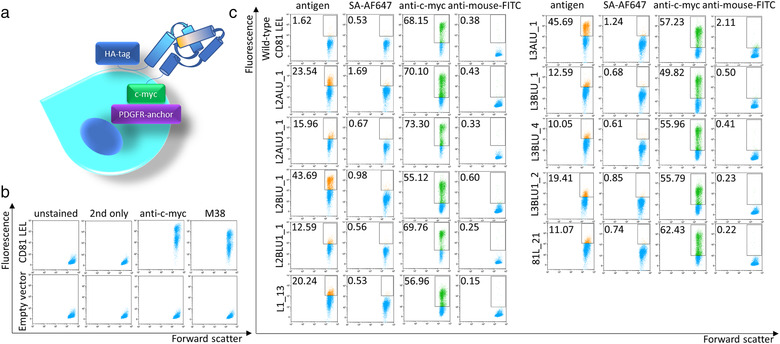
Screening of mutant CD81 LEL clones for laminin binding in mammalian expression system. (a) Schematic of surface display on HEK293 cells; (b) Display of wild‐type CD81 LEL; (c) Antigen binding and c‐myc epitope expression of selected laminin‐binding CD81 LEL variants. Figures indicate the percentage of gated cells. SA‐AF647: streptavidin‐Alexa Fluor 647

To increase the likelihood of antigen binding of selected CD81 LEL candidates, when expressed as full‐length CD81 on the surface of EVs produced by mammalian cells and to reduce the frequency of false‐positive hits, we expressed the mutant LELs of candidate clones in the pDisplay system in HEK293‐6E cells (schematic in Figure 3a). With this system, correctly folded wild‐type CD81 LEL can be displayed on the cell surface (Figure 3b) at copy numbers of 20,000 molecules per cell, as determined using QIFIKIT and an anti‐c‐myc tag antibody. The level of display was comparable for the wild‐type CD81 LEL construct and putative laminin‐binding clones. For nine mutants of yeast‐derived CD81 LEL, reactivity with 0.5 μM laminin could be established while no signal above the threshold staining was observed with secondary reagent only (Figure 3c). The five best clones were expressed as full‐length CD81 variants on the surface on EVs.

### 3.3 Production and characterization of recombinant EVs

**FIGURE 4:**
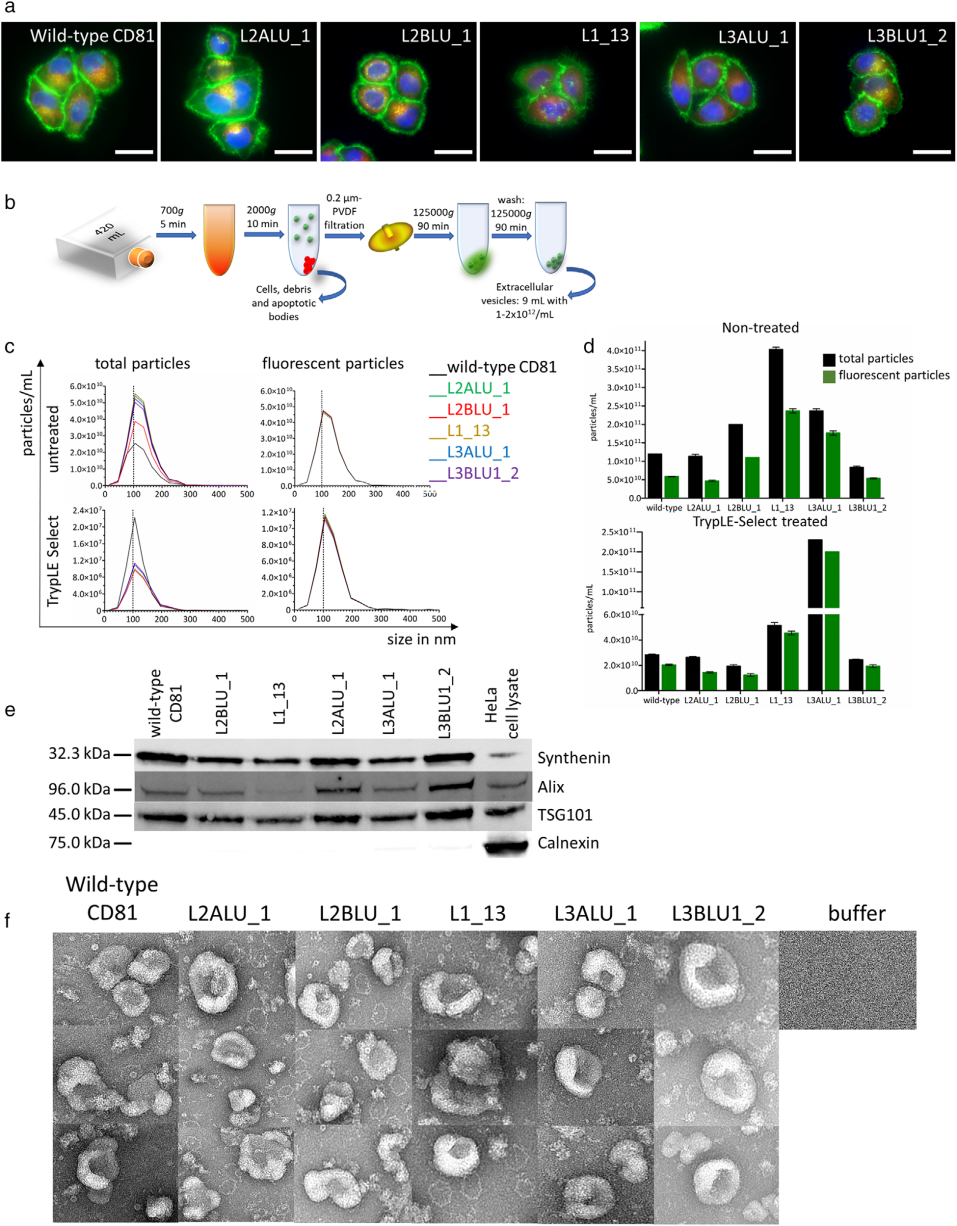
Production of EVs expressing antigen binding CD81 variants. (a) HeLa cells transformed with full‐length mutant CD81 analysed with live‐cell microscopy for distribution of recombinant CD81‐EGFP construct (green), Mitotracker (red) and Hoechst 33342 staining (blue) (bar represents 10 μm); (b) Scheme of EV enrichment; (c) Median size of isolated untreated and TrypLE‐Select‐ treated EVs produced by transformed cell lines (indicated in legend) for total and fluorescent particles; (d) Total (black bars) and recombinant EV (green bars) counts determined with NTA for untreated and TrypLE‐Select‐ treated EVs; (e) Western blot for EV marker proteins syntenin, Alix, TSG101, and the non‐EV marker calnexin; (f) TEM images of EVs isolated from wild‐type and mutant CD81‐transformed cells

Stable HeLa‐based cell lines, transduced with laminin‐binding CD81 LEL constructs cloned as full‐length CD81‐eGFP fusion, were found to express the mutant constructs at the same level as the analogous wild‐type variant and, similarly, the recombinant proteins predominantly localized to the cell membrane (Figure 4a and Figure S1). Each batch of isolated EVs (Schematic of EV preparation in Figure 4b) resulted in 1–2 × 10^12^ particles per ml with the median size between 100 and 125 nm as determined with NTA analysis, and there were no conspicuous differences between TrypLE‐Select treated and untreated EVs (Figure 4c and Figure S2A). Treatment of CD81‐expressing HeLa cells with TrypLE‐Select as applied here did not alter the surface expression of CD81 as opposed to 0.05% trypsin treatment (Figure S3). At least 40 % of the particles were eGFP‐fluorescent, indicating that they contained recombinant constructs (Figure 4d and Figure S2B). Importantly, a high yield of EV particles per cell was established for all mutants (Figure S2C). EV marker proteins syntenin, Alix and TSG101 were shown to be enriched in EV preparations in respect to HeLa cell lysate while calnexin, as a non‐EV protein, was absent (Figure 4e). TEM analysis confirmed the presence of the typical cup‐shaped structures (Figure 4f).

### 3.4 Cellular uptake of recombinant EVs and specific binding to laminin

**FIGURE 5:**
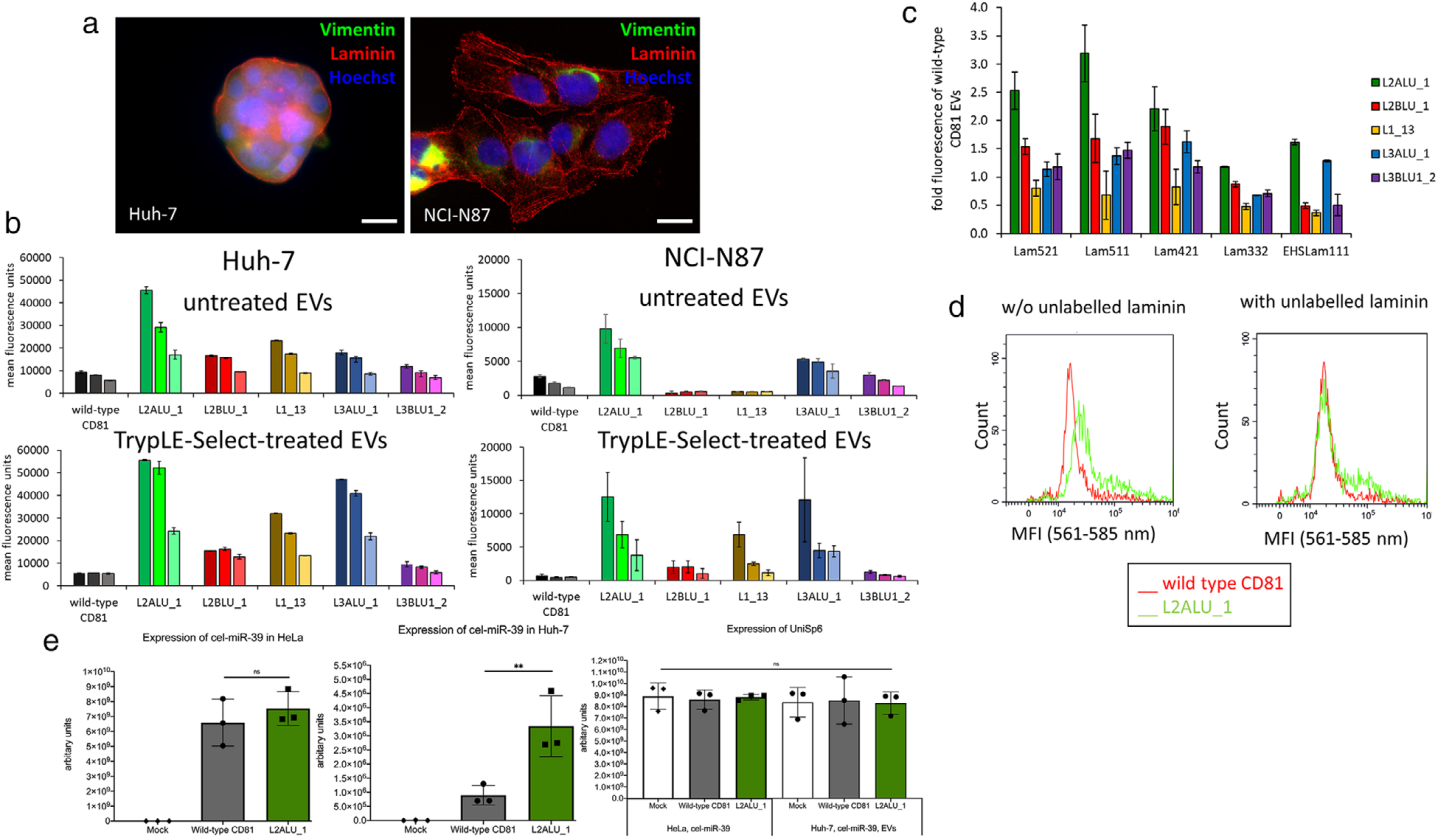
EVs expressing laminin‐binding CD81 internalize potently into laminin‐secreting cell lines. (a) Live‐cell microscopy images of Huh7 and NCI‐N87 cell lines visualizing laminin secretion (red), counterstained with anti‐vimentin (green) and Hoechst 33342 (blue) (bar represents 10 μm); (b) Internalization of laminin‐binding EVs into Huh‐7 and NCI‐N87 cell lines. For each clone, results of treatment with 2.5 × 10^10^ (dark colour), 1.25 × 10^10^ (medium dark colour) and 0.75 × 10^10^ (light colour) EVs are shown; (c) Binding of laminin isoforms (indicated in legend) to CD9‐bead captured recombinant EVs; (d) EVs with L2ALU_1 CD81 variant show specific binding of biotinylated laminin that can be outcompeted with excess of labelled antigen; (e) EV‐mediated transfer of cel‐miR‐39 to Huh‐7 cells. The levels of cel‐MiR‐39 are similar for EV‐producing HeLa cells transformed with wild‐type CD81 and L2ALU1 clone (left panel), but the expression in Huh‐7 cell line is significantly stronger after exposure L2ALU_1‐EVs (central panel). The levels of spike‐in RNA UniSp6 are similar for all cells and conditions (right panel). Individual measurements are presented as black bullets and 6 individual measurements were done for UniSp6 expression evaluation. Statistical analysis was done with one‐way ANOVA in GraphPad Prism version 5.03 (ns: *P* > 0.05; **: ≤ 0.01)

Laminin expression in Huh‐7 and NCI‐N87 cells was verified using live‐cell microscopy (Figure 5a and Figure S4). When Huh‐7 cells were treated with the highest tested concentration of EVs, most potent internalization was mediated by the clone L2ALU_1, where about five‐fold increase in fluorescence was measured in comparison with wild‐type CD81‐EV treated cells (Figure 5b). When the cells were exposed to TrypLE‐Select‐treated EVs, the difference amounted even to 9.9‐fold. Similar behaviour was observed for the clone L3ALU_1 where the fold change in fluorescence in respect to wild‐type increased from 1.9 to 8.4‐fold, and for L1_13 where 2.5‐ to 5.7‐fold increase could be recorded. For the other candidate binders, the increase in fluorescence was less prominent, 1.8‐ versus 2.7‐fold with TrypLE‐Select reagent treatment for the L2BLU_1 clone, and 1.2‐ and 1.7‐fold for the L3BLU1_2 clone. Dose‐dependent increase in fluorescence regarding the concentration of EVs used was observed for most assayed clones and conditions, but most prominently for TrypLE‐Select treated EVs expressing clones L2ALU_1, L3ALU_1 and L1_13.

Most promising CD81 variants were also able to enhance the internalization into the NCI‐N87 cell line, 3.5‐ and 1.9‐fold increase in fluorescence signal could be measured for cells exposed to L2ALU_1 and L3ALU_1‐harboring EVs compared to wild‐type CD81 EVs. For this cell line, the effects with TrypLE‐Select treated EVs were stronger, with 17.5‐ and 16.8‐fold increase in fluorescence with clones L2ALU_1 and L3ALU_1. With this protocol a 9.6‐fold increase was observed with L1_13 clone and a 2.7‐fold increase with L2BLU_1 clone, while in both cases only background fluorescence levels were recorded when the EVs were not pretreated. The fluorescence of the cells treated with L3BLU1_2‐EV variant was at the level of wild‐type CD81 EV‐treated cells, regardless of the pre‐treatment protocol.

To corroborate the enhanced uptake of recombinant EVs, biological replicates of the preparations L2ALU_1 and L3ALU_1 were tested for the uptake into Huh‐7 and NCI‐N87 cells and the fold change in fluorescence in respect to wild‐type increased for about two‐ to three times. The enhanced EV internalization of TrypLE‐Select treated EVs was observed as a six‐ to eight‐fold increase in fluorescence compared to wild‐type EVs in both cell lines (Figure S5).

Recombinant EVs were tested for their reactivity with several variants of biotinylated laminin and the potently internalizing EVs with clone L2ALU_1 showed more than two‐fold increase in response when tested for reactivity with recombinant laminin variants with γ1 subunit, as well as a 1.5‐fold response with NHS‐laminin111, in comparison with wild‐type EVs (Figure 5c). EVs, displaying other clones, were within 1.5‐fold of the response of the wild‐type EVs.

To examine if the interaction of the recombinant EVs with the target antigen was specific, we performed a flow cytometry‐based assay where the EVs harbouring either wild‐type CD81‐GFP or the CD81 variant L2ALU_1 were first captured to anti‐CD9 coated beads and allowed to react with biotinylated human placental laminin and neutravidin‐PE. A higher level of fluorescence was seen for the L2ALU_1‐EVs than for wild‐type CD81‐EVs (Figure 5d). Specificity of interaction was tested in an experiment using competition with an excess of unlabelled laminin, which indeed reduced the amount of labelled laminin bound by L2ALU_1 clone.

### 3.5 EV‐packaged cel‐miR‐39 transfer

To assess the enhanced internalization of CD81 variants, HeLa cells stably expressing wild‐type CD81‐GFP or the CD81 variant L2ALU_1 were reversely transfected with *C. elegans* cel‐miR‐39 precursor (Terlecki‐Zaniewicz et al., 2019). Seventy‐two hours after transfection, EVs were isolated and used for Huh‐7 cells treatment. After 24 h of exposure, Huh‐7 small RNA was isolated and transfer of cel‐miR‐39 was analysed using qPCR. The expression of cel‐miR‐39 in EV‐producing HeLa cells was similar for wild‐type CD81‐transfected cells and L2ALU_1 variant. Remarkably, cel‐miR‐39 was detected in recipient Huh‐7 cells, and the levels were found to be significantly increased in Huh‐7 cells exposed to L2ALU_1‐EVs compared to wild‐type CD81 EVs, while levels of UniSp6 spike‐in were comparable for all cells and conditions (Figure 5e). Furthermore, the effect of the targeted EVs was lost when EVs were treated with Triton X‐100 and RNase A before exposing them to recipient Huh‐7 cells (Figure S6).

## 4 DISCUSSION

Stem cell therapies have been applied for the regeneration of a wide range of injured or lost tissues, but only recently it has been proposed that the therapeutic effects of stem cells largely depend on their paracrine activity and are mediated by growth factors and EVs (Azoidis et al., 2018). The advantage of applying EVs rather than growth factors is that they deliver a complex cargo of physiologically relevant proteins and nucleic acids and can even be loaded with exogenous bioactive substances. Comparing with cells, vesicles cannot replicate, do not change their phenotype and are more likely to reach the target tissue due to their small size. Specific targeting of EVs appears to be one of the challenging tasks to be accomplished before these efficient intercellular mediators can be widely used as therapeutic agents, and this has been approached with several strategies involving membrane surface decoration or alteration of their membrane composition by manipulation of their source cell (Murphy et al., 2019). We attempted here to enhance the interaction of extracellular vesicles with their target antigen by assigning one of the overrepresented EV‐membrane marker proteins, CD81, the ability of specific antigen recognition. The actual scaffold for directed evolution were thermally stabilized variants of the CD81 LEL with a high T_M_ over 90°C, which is regarded prerequisite for randomization at a level required to achieve strong antigen binding. The amino acid residues, proposed for randomization, have solvent‐exposed side chains and should together form a confluent surface, which, in size, is comparable with antigen‐binding surfaces of clinically‐relevant alternative antigen‐binding scaffolds V_H_H that have already been shown to mediate high‐affinity antigen interaction, even though they are of smaller molecular surface areas and smaller diameters than conventional antibodies. The large extracellular loop of CD81 was well expressed in yeast display system, which triggered the choice of this platform. Furthermore, it has already served as a selection tool, not only for antibodies (Boder et al., 2012) and other immunoglobulin domain‐based binders, but also unrelated proteins of various architectures (Könning & Kolmar, 2018), such as Fibronectin 10th Type III domain (Koide et al., 1998), Sso7d (Gera et al., 2011) and Gp2 (Kruziki et al., 2015).

The yeast‐displayed libraries, based on the designed CD81 LEL scaffolds, were constructed with a high percentage of correct members, exhibited a high level of display of mutated proteins, and were rapidly enriched for the antigen of choice, human placental laminin. As the envisioned protocol for antigen‐binding EVs production involved the time‐consuming construction of stably transformed cell lines, a simple in‐between screening step of selected variants of CD81 LEL with mammalian surface display was introduced. Here the mutated LEL domains were expressed as C‐terminal fusions as opposed to the N‐terminal constellation in yeast display, and their expression was additionally under strict quality control of the mammalian folding machinery. Nevertheless, reactivity with the antigen could be corroborated for all variants enriched in yeast display, and the five, with the highest level of antigen binding, were expressed as GFP‐fused full‐length proteins in HeLa cells.

As the level of their expression as well as membrane‐localization was similar to the wild‐type CD81 construct, preparations of the derivatized EVs were compared for the ability to internalize into laminin‐secreting cell lines, Huh‐7 and NCI‐N87. Vesicles isolated from all host cell lines contained a high proportion of GFP‐positive entities and displayed similar uniform size distribution, with majority of the particles measuring 100 nm in diameter, which is typical for EVs. An increased uptake into two target laminin‐secreting cell lines was established for four out of the five selected EVs, and this feature was even more prominent after the pre‐treatment of EV preparation with TrypLE‐Select reagent. This protocol step was employed to digest away the laminin fragments present in the EV preparation and hence minimize the potential scavenging of the available binding sites, and additionally reduce the hydrodynamic radius of EVs by thinning of their corona zone (Skliar et al., 2018), which reportedly impedes their migration rate. The interaction of the most potently internalizing clone L2ALU_1 with laminin was shown to be specific upon competition with unlabelled laminin, and the potentiated reactivity with recombinant laminin variants 521, 511, 421, and EHS laminin111 indicated its specific binding to the γ1 laminin subunit. Importantly, the cel‐miR‐39 delivery into target cells was enhanced using L2ALU_1 derived EVs in comparison with wild‐type CD81 expressing EVs, corroborating the superior internalization of the target‐specific vesicles.

Laminins are major signalling and structural molecules of basement membranes and modulate several diverse cellular functions, such as maintaining tissue structure, adhesion and migration (Aumailley, 2013), but also cell polarity, survival and hormone signalling (Domogatskaya et al., 2012; Hohenester & Yurchenco, 2013; Xu et al., 2010). Laminin internalization could be mediated either by receptor‐independent mechanisms such as pinocytosis, or receptor‐dependent processes. Deficient endocytosis of laminins by dystroglycan results in a dysfunctional basement membrane, perturbed signalling from the endocytic compartment and affects laminin‐associated proteins, indispensable to normal cellular regulation (Leonoudakis et al., 2014). Another receptor connected with laminin endocytosis is the nonintegrin type 67 kDa laminin receptor (67LR). Laminin‐mediated internalization of this receptor can even be neuroprotective as it inhibits entry of β‐amyloid peptide into neurons, prevents its accumulation and decreases neurotoxicity (Gopalakrishna et al., 2018). There is accumulating evidence that EVs are bound to the molecules within the extracellular matrix, in part via ligand‐receptor interactions (Hoshino et al., 2015; Sung et al., 2015), and can in this form modulate cell migration and invasiveness (Mu et al., 2013) as well as mediate regulated delivery of molecular cargo (Clancy et al., 2015). Taken together, these facts point into a direction that EVs, with enhanced laminin binding, may efficiently support regenerative processes.

In summary, we have established a novel selectable antigen binding platform based on the CD81 LEL scaffold. The mutant laminin‐binding CD81 LEL sequences, derived from yeast display, were confirmed to interact with the cognate antigen, and the resulting EVs have indeed shown a higher level of internalization into laminin‐secreting cell lines, including the ability of potent cel‐miR‐39 transfer. In the view of the broad scope of applications for targeted EV‐based therapies, the advantage of the method presented here is that it can rapidly deliver binders to any antigen of choice, which can simply be ‘clicked’ into the full‐length CD81, recombinantly expressed on the EV surface, enabling specific EV‐mediated delivery to a large variety of cells and tissues.

## CONFLICT OF INTEREST

J.G. is co‐founder and shareholder of Evercyte GmbH. The technology of antigen recognition via antigen binding marker proteins of extracellular vesicles of is covered in the patent application PCT/EP2019/071825.

## Supporting information

Supporting information.

Supporting information.

Supporting information.

Supporting information.

Supporting information.

Supporting information.

Supporting information.

Supporting information.

## Data Availability

The data supporting the findings of this study are available within the article and its supplementary materials.
