# Supplementary figures and images for: An engineered CD81‐based combinatorial library for selecting recombinant binders to cell surface proteins: Laminin binding CD81 enhances cellular uptake of extracellular vesicles
Source: J Extracell Vesicles. 2021 Sep 12;10(11):e12139. doi: 10.1002/jev2.12139 (PMC8435527; doi:10.1002/jev2.12139)

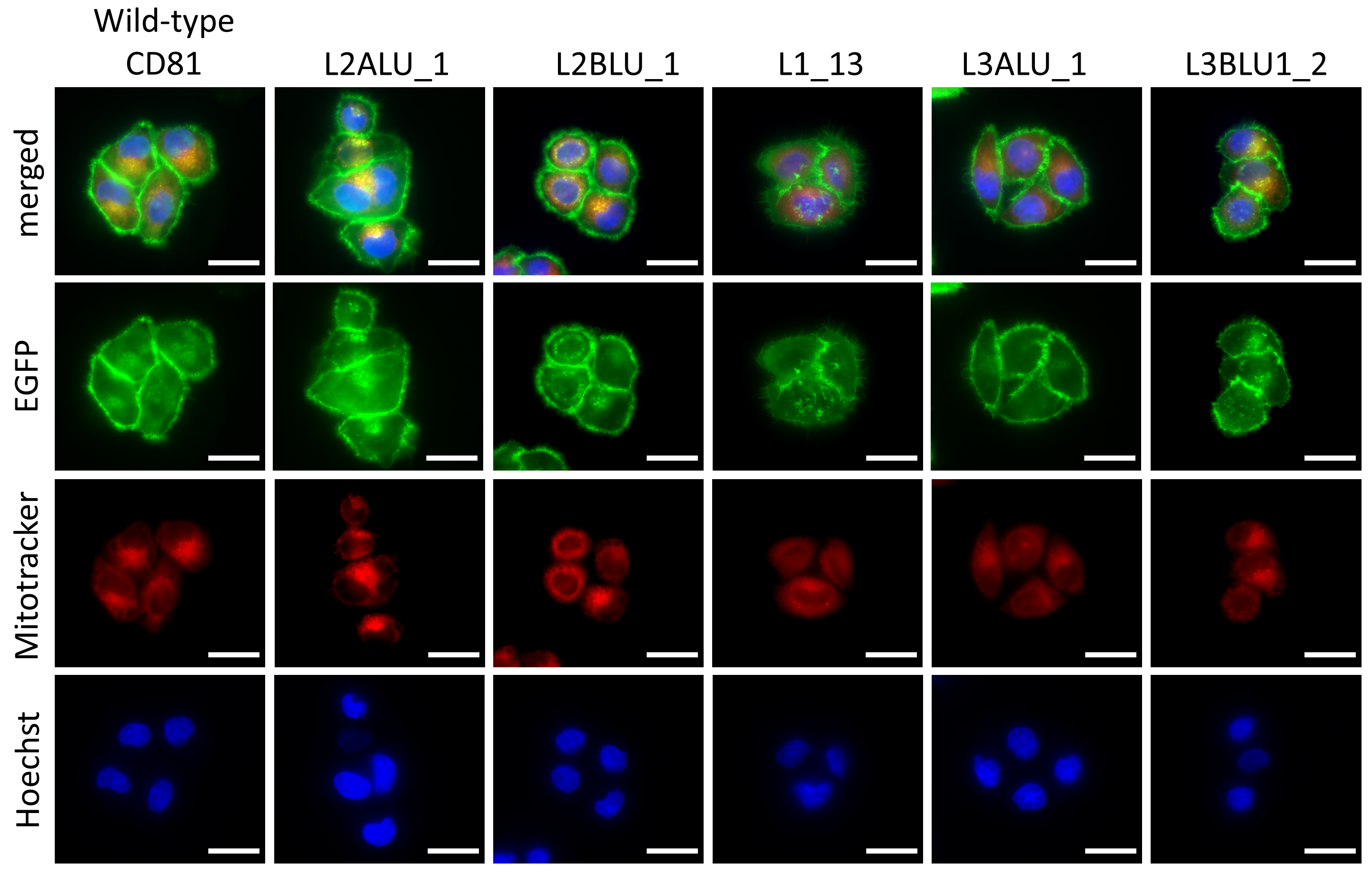

Supplement: Supplementary file 1 — Supporting information. [file JEV2-10-e12139-s003.tif]

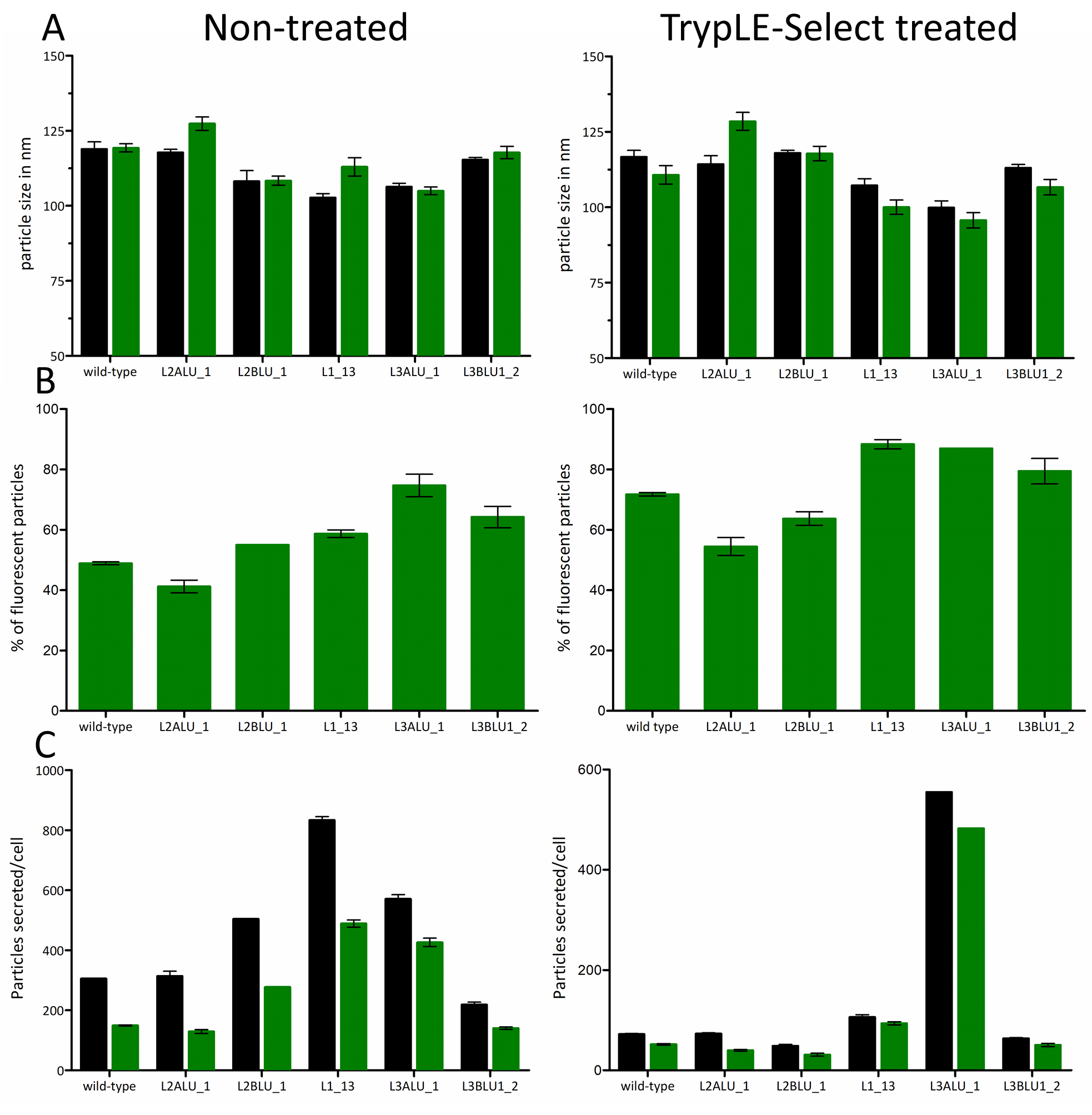

Supplement: Supplementary file 2 — Supporting information. [file JEV2-10-e12139-s004.tif]

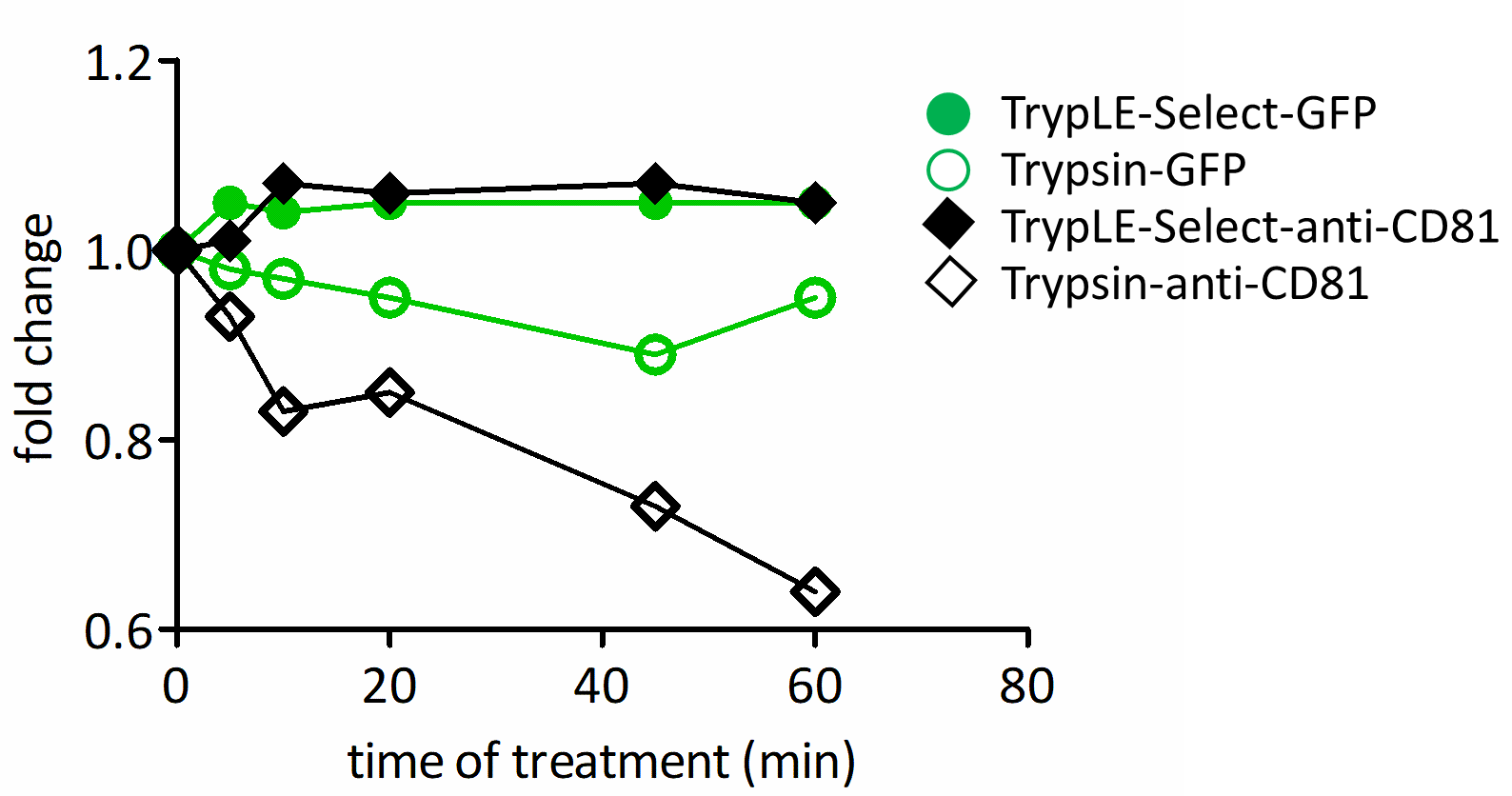

Supplement: Supplementary file 3 — Supporting information. [file JEV2-10-e12139-s008.tif]

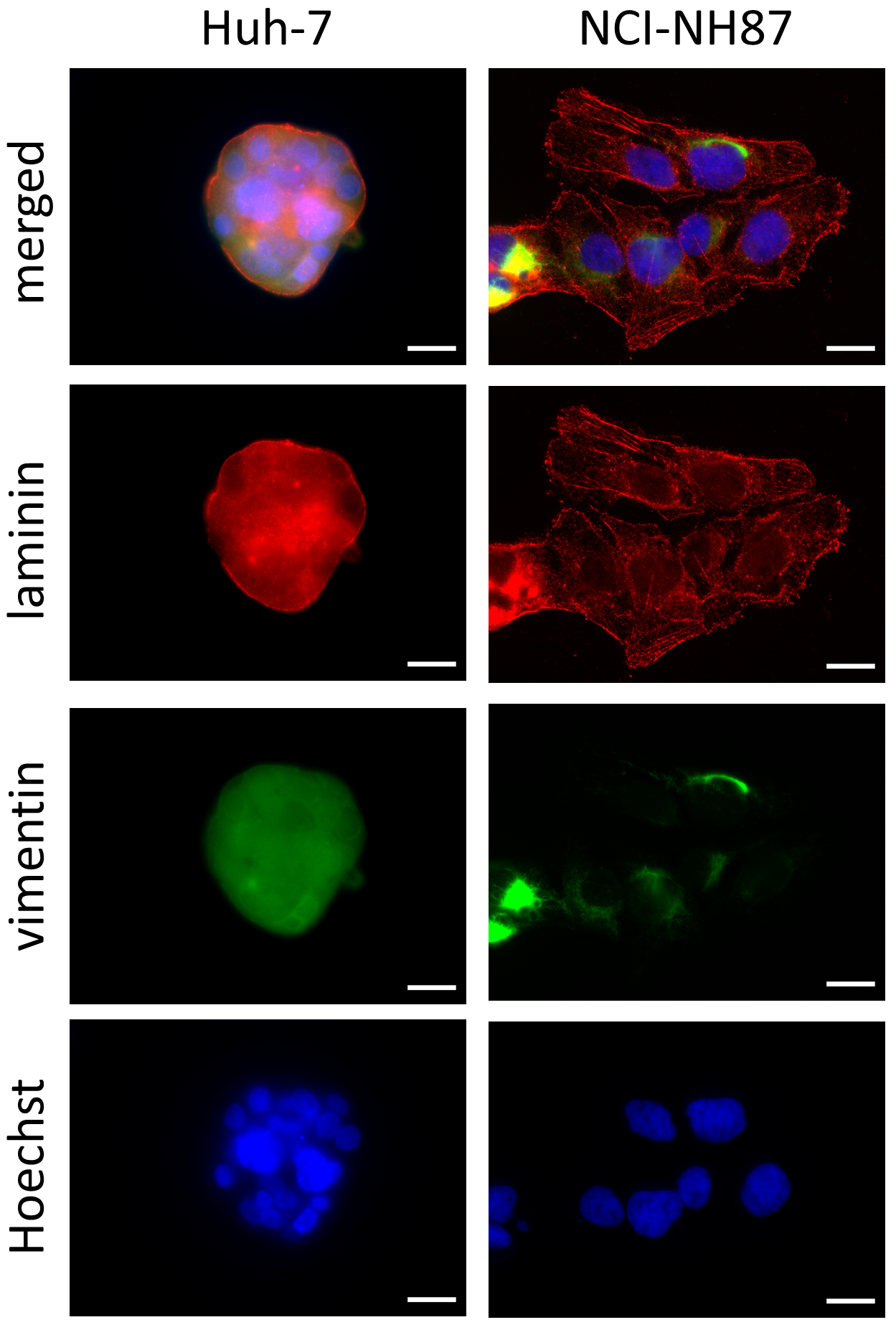

Supplement: Supplementary file 4 — Supporting information. [file JEV2-10-e12139-s007.tif]

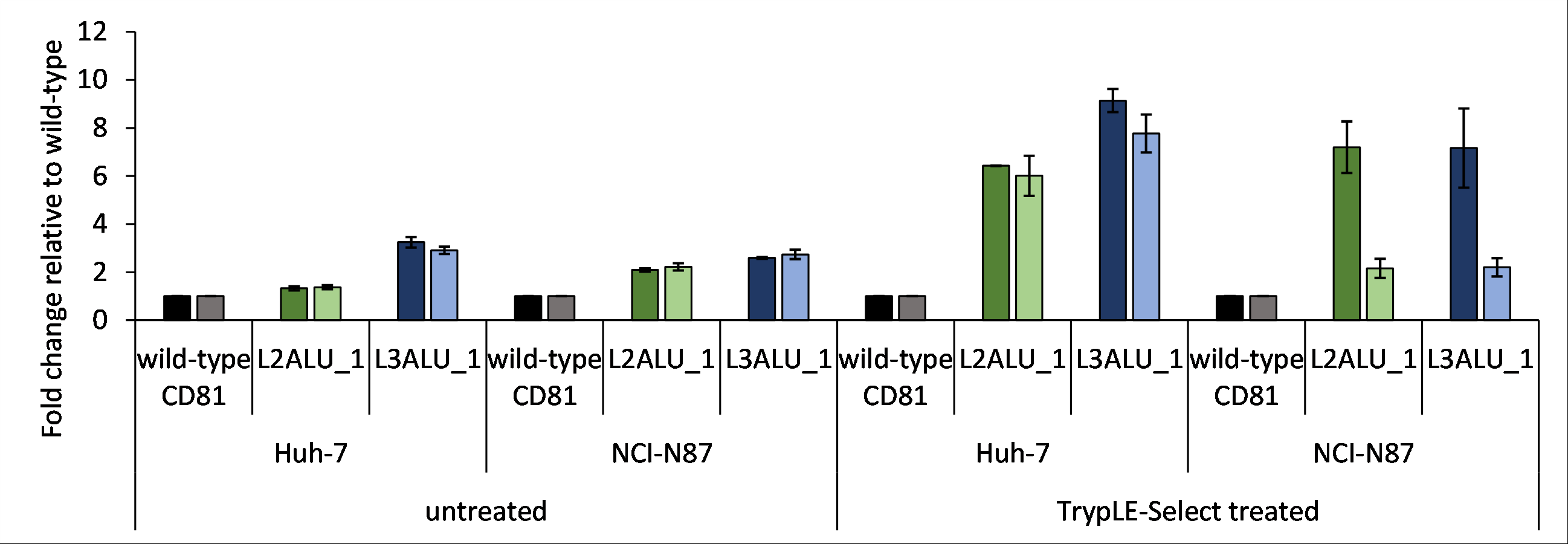

Supplement: Supplementary file 5 — Supporting information. [file JEV2-10-e12139-s005.tif]

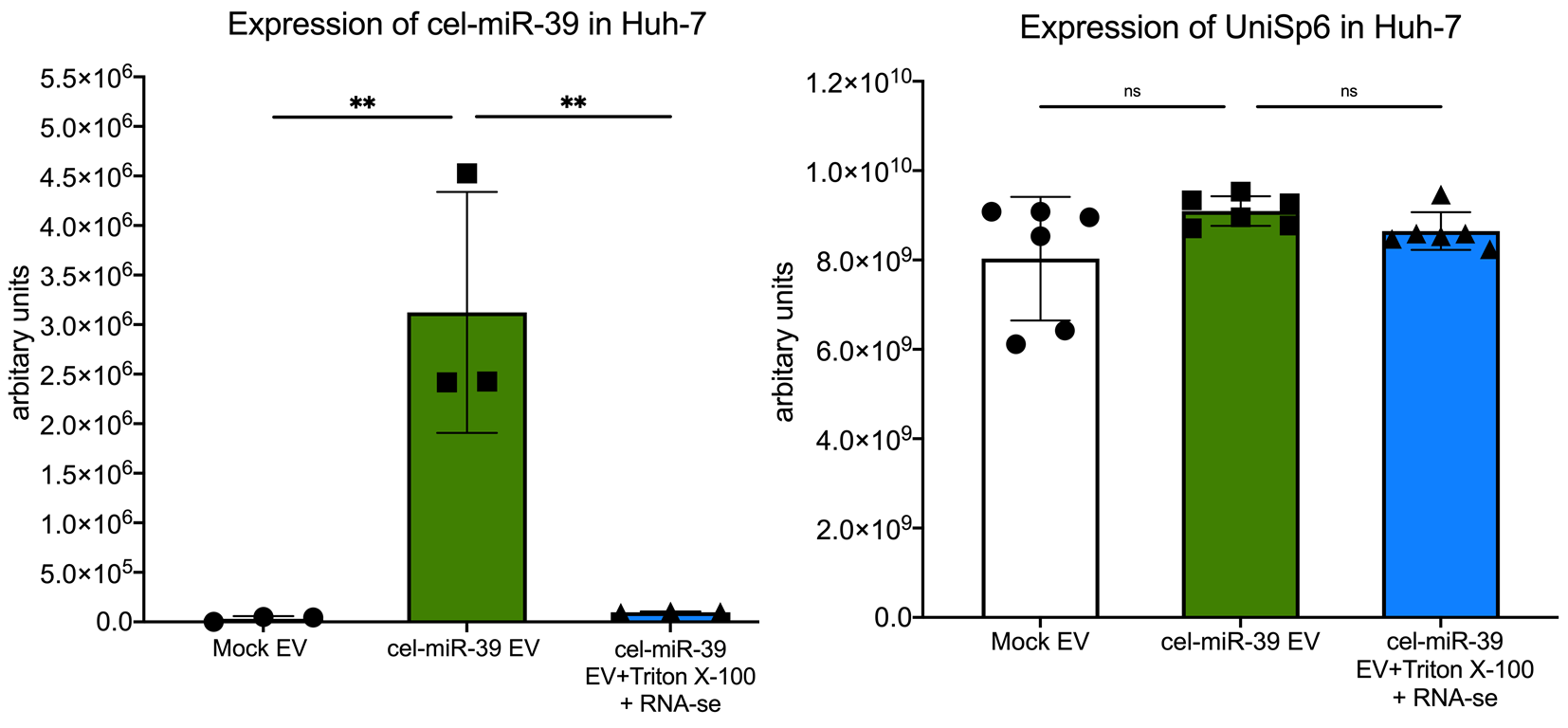

Supplement: Supplementary file 6 — Supporting information. [file JEV2-10-e12139-s002.tif]
