# Supplementary material for: An engineered CD81‐based combinatorial library for selecting recombinant binders to cell surface proteins: Laminin binding CD81 enhances cellular uptake of extracellular vesicles
Source: J Extracell Vesicles. 2021 Sep 12;10(11):e12139. doi: 10.1002/jev2.12139 (PMC8435527; doi:10.1002/jev2.12139)
Supplement: Supplementary file 7 — Supporting information. [file JEV2-10-e12139-s001.docx]

Supplementary Table 1: Oligonucleotide sequences

| Cloning of CD81 into pyd1 | |
| --- | --- |
| CD81ydbam1 | acgtggatcctttgtcaacaaggaccagatc |
| CD81ydnot2 | acgtgcggccgcgccttcccggagaagaggtc |
| Mutagenesis of pYD1_CD81 library vector | |
| 81delhind | gtatgtttttaagctcctgcaggctagtggtg |
| 81delhinda | caccactagcctgcaggagcttaaaaacatac |
| 81libbam | tttgtgtccctcgggatccaacatcatcagcaa |
| 81libbama | ttgctgatgatgttggatcccgagggacacaaa |
| 81libhind | gcagttctatgaccaagctttacagcaggccgtgg |
| 81libhinda | ccacggcctgctgtaaagcttggtcatagaactgc |
| CD81 library construction | |
| L2for | aaggatgtgaagcagttctatgaccaagctttannknnktgctgtnnknnknnknnkgccaacaacgcctgtgctgtg |
| L3for | aaggatgtgaagcagttctatgaccagtgtctannknnkgcctgtnnknnknnknnknnknnkaacgccaaggcttgtgtg |
| EFrev | cttgaagaggttgctgatgatgttggatcccgagggacacaaattmnnmnngagcacacamnnggtmnnmnnmnnmnntgtgctggagccacagca |
| Sequencing of pYD1-cloned variants | |
| pyd forward | agtaacgtttgtcagtaattgc |
| pyd reverse | gtcgattttgttacatctacac |
| Cloning of CD81 into pDisplay | |
| CD81dissfi1 | acgtggcccagccggcctttgtcaacaaggaccag |
| CD81dissal2 | acgtgtcgaccttcccggagaagaggtcatcg |
| Sequencing of pDisplay-cloned variants | |
| T7forward | taatacgactcactataggg |
| pdisrev | gctgagatcaccaccacc |
| Cloning of full-length CD81 into pBMN-I-GFP | |
| CD81-into-pBMN-fwd | ccaccggtcgccaccatgggagtggagg |
| CD81-into-pBMN-rev | atcgtcgacgcggccgatgtacacggagctgtttcgaatgcc |
| Sequencing of pBMN-I-GFP and of pBMN-I_GFP_CD81 mutants | |
| pBMN-seq-fwd | gatacacgccgcccac |
| pBMN-seq-rev | caggtggggtctttcattcc |
| Cloning of mutant LELs into pBMN-I-GFP_CD81 | |
| 81LELopenfwd  81LELopenrev  81LELfwd | ctgtacctcatcggcattgctgc  gccccagatgccggcg  gccggcatctggggctttgtcaacaaggaccagatcg |
| 81LELrev | gccgatgaggtacagcttcccggagaagaggtcatcg |
