# Supplementary material for: An engineered CD81‐based combinatorial library for selecting recombinant binders to cell surface proteins: Laminin binding CD81 enhances cellular uptake of extracellular vesicles
Source: J Extracell Vesicles. 2021 Sep 12;10(11):e12139. doi: 10.1002/jev2.12139 (PMC8435527; doi:10.1002/jev2.12139)
Supplement: Supplementary file 8 — Supporting information. [file JEV2-10-e12139-s006.docx]

Supplementary File 1

Cloning of full-length CD81 variants for the expression in mammalian cells

The coding sequence of full-length wildtype CD81 was inserted into pBMN-eGFP by using the In-Fusion cloning system (Takara). The backbone plasmid pBMN was digested via BamHI and NotI and fused with the PCR-amplified wildtype full-length CD81 insert derived from pDisplay (CD81-into-pBMN-fwd/CD81-into-pBMN-rev). The resulting plasmid pBMN-CD81-eGFP served as backbone for the cloning of mutated library-derived LEL sequences. Plasmid was linearized using inverse PCR with the primers 81LELopenfwd and 81LELopenrev. Mutated CD81 sequences were amplified using 81LELfwd and 81LELrev oligonucleotides and subsequently fused with linearized pBMN-CD81-eGFP via In-Fusion cloning.

Fluorescence microscopy to confirm membrane localization of CD81-GFP constructs

1.5 x10^4^ HeLa cells expressing CD81-eGFP variants were inoculated in 300 µL of RPMI 1640 medium (Sigma-Aldrich), supplemented with 10% fetal bovine serum (FBS) (Sigma-Aldrich), and 4 mM L-glutamine (Thermo Fisher Scientific) and seeded into the chambers of an 8-well glass culture slide (Ibidi). After a 48-h incubation at 37 °C in humidified atmosphere with 5% CO_2_, culture medium was removed from each well and replaced with Live Cell Imaging Solution (Thermo Fisher Scientific) containing 500 nM MitoTracker® probe (Thermo Fisher Scientific), prewarmed to 37 °C and incubated for 30 min at 37 °C. The samples were washed three times with ice-cold PBS, fixed with 3.5% paraformaldehyde in PBS pH 7.4 for 5 min on ice and 5 min at RT. Fixation reagent solution was discarded, cells were washed three times with PBS and then stained with 5 µg/mL Hoechst 33342 for 5 min, washed again with PBS and analyzed by fluorescence microscopy on a Leica DMI-6000B microscope equipped with filter sets for DAPI, GFP and RFP.

TrypLE-Select reagent treatment of HeLa cells

To test the effect of TrypLE-Select reagent treatment on the stability of cell/EV surface epitope expression, HeLa cells stably expressing CD81-eGFP variant L2ALU_1 were treated with either 0.05 % trypsin or TrypLE-Select agent for 0, 5, 10, 20, 45 and 60 min at 37°C. After this step, cells were centrifuged at 300*g* for 5 min to remove the enzyme and washed once with ice-cold PBS. The surface levels of CD81 on live cells were quantified by staining with anti-CD81-APC antibody (130-119-825; Miltenyi Biotec) at 1:50 dilution and the level of green fluorescent protein (GFP) signal: flow cytometry was used to record the mean fluorescent intensity values of the main cell population, gated out in the FSC/SSC plot, using a CytoFLEX S instrument (Beckman Coulter).

Fluorescence microscopy to confirm expression of laminin

1.8 x10^4^ Huh-7 and 2.4 x10^4^ NCI-N87 cells were inoculated in 300 µl of high glucose DMEM (Thermo Fisher Scientific), supplemented with 10% fetal bovine serum (Sigma-Aldrich), and 4 mM L-glutamine (Thermo Fisher Scientific) and seeded into the chambers of an 8-well glass culture slide (Ibidi). After a 48-h-incubation at 37 °C in humidified atmosphere with 5% CO_2_, culture medium was removed from each well and cells were washed three times with ice-cold PBS and fixed with 300 µL of 4% paraformaldehyde in PBS pH 7.4 per well, 5 min on ice and 5 min at RT. Cells were again washed three times with ice-cold PBS and permeabilized with 0.15% of Triton-X in PBS pH 7.4 for 10 min. After washing three times with ice-cold PBS, samples were incubated with 1% BSA, 22.52 mg/mL glycine in PBST (PBS+ 0.1% Tween-20) for 30 min to block unspecific binding of the antibodies. Primary antibody reactive with laminin γ-chain (HPA-001909, Sigma-Aldrich, rabbit polyclonal) was applied in a 1:50 dilution with the blocking solution for 1 h at RT. After a washing step with PBS, cells were incubated with the secondary detection antibody (anti-rabbit-DyLight 594, 35560, Thermo Fisher Scientific) in a 1:500 dilution in blocking solution with or without anti-vimentin antibody conjugated to Alexa Fluor® 488 (clone RV202, 562338, BD Biosciences) as cytoskeleton marker. Finally, the samples were washed three times with PBS, stained with 5 µg/mL Hoechst 33342 for 5 min, washed once with PBS and analyzed by fluorescence microscopy on a Leica DMI-6000B microscope equipped with filter sets for DAPI, GFP and RFP.
